# Classification of Complete Proteomes of Different Organisms and Protein Sets Based on Their Protein Distributions in Terms of Some Key Attributes of Proteins

**DOI:** 10.1155/2018/9784161

**Published:** 2018-03-04

**Authors:** Hao-Bo Guo, Yue Ma, Gerald A. Tuskan, Xiaohan Yang, Hong Guo

**Affiliations:** ^1^Department of Biochemistry and Cellular and Molecular Biology, University of Tennessee, Knoxville, TN 37996, USA; ^2^Biosciences Division, Oak Ridge National Laboratory, Oak Ridge, TN 3783, USA

## Abstract

The existence of complete genome sequences makes it important to develop different approaches for classification of large-scale data sets and to make extraction of biological insights easier. Here, we propose an approach for classification of complete proteomes/protein sets based on protein distributions on some basic attributes. We demonstrate the usefulness of this approach by determining protein distributions in terms of two attributes: protein lengths and protein intrinsic disorder contents (ID). The protein distributions based on *L* and ID are surveyed for representative proteome organisms and protein sets from the three domains of life. The two-dimensional maps (designated as fingerprints here) from the protein distribution densities in the LD space defined by ln(*L*) and ID are then constructed. The fingerprints for different organisms and protein sets are found to be distinct with each other, and they can therefore be used for comparative studies. As a test case, phylogenetic trees have been constructed based on the protein distribution densities in the fingerprints of proteomes of organisms without performing any protein sequence comparison and alignments. The phylogenetic trees generated are biologically meaningful, demonstrating that the protein distributions in the LD space may serve as unique phylogenetic signals of the organisms at the proteome level.

## 1. Introduction

Determination of complete genome sequences for a number of organisms has offered an unprecedented opportunity for biological community and transformed biology into a discipline that depends significantly on how to classify and interpret large-scale data sets and to extract biological insights from these data sets. The traditional ways of thinking and approaches from the pregenomic era (e.g., the sequence comparison/alignment and homology identification) are of fundamental importance in the postgenomic era. Nevertheless, new approaches based on some global features of omics data sets need to be explored in order to make classification and comparison of large-scale data sets easier. For proteomes, this may be achieved, for instance, through identification of key parameters or attributes of proteins and comparison of protein distributions within complete proteomes of different organisms or protein sets in terms of such parameters or attributes.

In this paper, we adapt this approach and use two parameters of proteins for the purpose of classifying complete proteomes of different organisms (for simplicity, proteomes) and protein sets: the length of protein amino acid (aa) sequence (protein length *L* hereafter) and intrinsic disorder content (protein disorder ID hereafter). It had been proposed that the protein sizes, folding rates, and many other physical properties could be associated or even determined by *L* [1, 2]. At the level of proteomes, previous studies have suggested that the eukaryotic proteomes may exhibit averagely longer *L* compared to the prokaryotic proteomes [3, 4], even though further analysis may still be necessary. The importance of intrinsically disordered proteins (IDPs) and protein regions (IDPRs) has been recognized [5–13], and it has been observed that relatively high contents of intrinsic disorders may exist for eukaryotic proteins than for prokaryotic proteins [14]. Moreover, proteins expressed in two eukaryotic organelles, chloroplasts and mitochondria, which evolved from cyanobacteria and alphaproteobacteria, respectively, seem to have a lower disorder content, on average, compared to nuclear-encoded proteins in their host eukaryotes [15]. Interestingly, it has been demonstrated that intrinsically disordered proteins are associated with a variety of human diseases [16, 17], including cancers [18, 19]. As a result, intrinsically disordered proteins have become important targets for drug design [20–25]. Thus, understanding intrinsically disordered proteins at the proteomic levels would be of considerable interest. The observations that the distributions of proteins in terms of ID and *L* may be different for proteomes and for different protein sets suggest that such distributions may be used to classify proteomes of different organisms or protein sets. They may also be used in the future to help understand the properties of proteomes in different disease states, as there seems to be a wide variability of predicted disorder among different diseases [26]. It is interesting to see that a recent study revealed that the overall disorder fractions are positively correlated to the size of the proteomes (estimated by the total aa numbers) and that the disorder fractions of the proteomes of large bacteria (more than 2.5 M aa) are comparable to those of eukaryotes [27].

**Figure 1:**
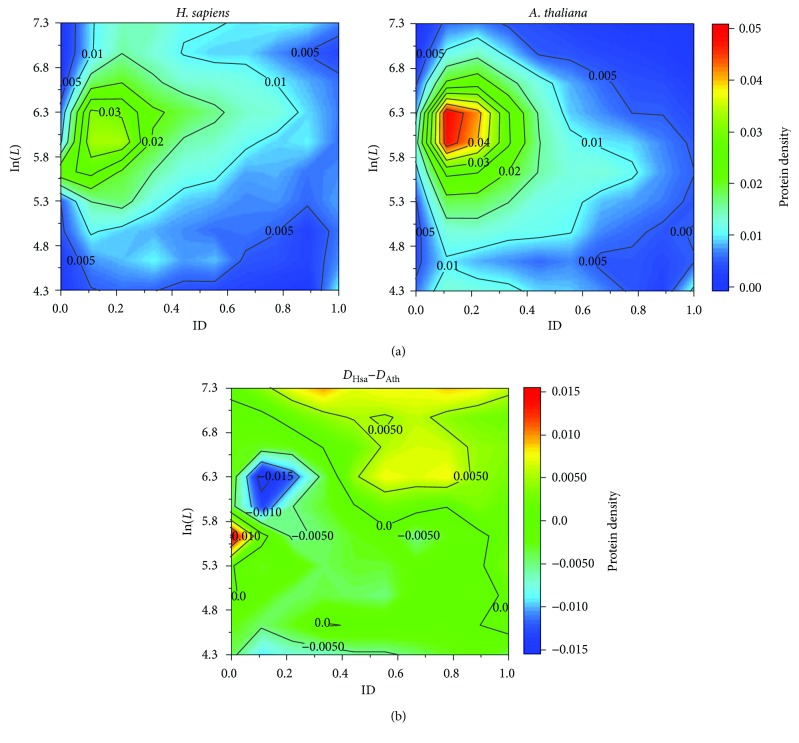
(a) Representative protein-density contour maps of (left) an animal (*H. sapiens*) and (right) a plant (*A. thaliana*) proteome. Short proteins (ln(*L*) < 4.3 or *L* < 74) and long proteins (ln(*L*) > 7.3 or *L* > 1480) are treated as ln(*L*) = 4.3 and ln(*L*) = 7.3, respectively, for statistics. (b) Differential protein density contour map between *H. sapiens* (*D*_Hsa_) and *A. thaliana* (*D*_Ath_) indicates that short disordered proteins are enriched in the plant proteome; and the animal proteome has more long disordered proteins.

Here we analyze the protein distributions in terms of *L* and ID from proteomes of different organisms across the three domains of life, collective data sets of organelles (plasmids, chloroplasts, and mitochondria), and the proteome data of two giant DNA viruses (termed giruses in literature). We noticed that the eukaryotic proteomes do not always exhibit averagely longer proteins than the prokaryotic proteomes. Our observation on protein disorder agrees well with the previous finding, that is, the average disorder contents in eukaryotic proteins are indeed higher than those in prokaryotic proteins. The two-dimensional maps (designated as fingerprints here) based on the protein distribution densities in the LD space defined by ln(*L*) and ID for the representative proteomes of different organisms and protein sets were constructed, and these fingerprints show distinct patterns for different organisms and protein sets. The features and relationships among the fingerprints are analyzed and compared. To test if our classification of proteomes of different organisms and protein sets proposed here is meaningful, we generated phylogenetic trees based on the protein distribution densities in the fingerprints of proteomes of different organisms without performing any protein sequence comparison and alignments. The phylogenetic trees generated in this way were found to be meaningful, as they contain important information of evolution. Thus, the proposed approach may represent a useful and simple way for proteome classification and comparison. In present study, for each protein-encoding gene locus only the prime protein has been used, therefore, the protein densities (Figure 1 and Figure S1) could be regarded as the gene densities. Moreover, using the poplar proteome as an example, it was found that the phylogenies show little difference with or without using alternative splicing proteins (Figure S3). Discussions are made concerning the possibility for extending this approach through introduction of additional attributes.

## 2. Results

### 2.1. Protein Distributions in Terms of *L* and ID

Here, we discuss the proteins (811,600 entries in total) from the proteomes of different organisms and protein sets listed in Table 1, with the protein lengths varying over three degrees of magnitude from 5 (*Os06g47230* of rice) to 34,350 aa (*titin* of human). For the protein length comparison, as pointed out previously [4], the median length is a better measure than the average length to avoid biases from extremely long proteins. Table 1 lists both the median and average lengths of all the proteomes and proteins from gene sets. It should be pointed out that in the present analysis, only the primary protein at each gene locus is selected. This allows a significant simplification of proteome classification. This approximation seems to be reasonable for the main purpose of this work, as there is little difference in the results for the test cases with or without using alternative splicing proteins. Table 1 shows that the eukaryotic proteomes do not *always* have averagely longer proteins than those in the prokaryotic proteomes, as previously suggested [3, 4]. For instance, the basal flowering plant *Amborella trichopoda* has a median protein length of 218, shorter than all prokaryotes (Archaea and bacteria) surveyed here. In addition, *Giardia intestinalis* in the Eukaryota domain has an even shorter median protein length of 147. The average *L*s show the same trend as the median values (Table 1).

Nevertheless, the proteins in a eukaryotic proteome do have a significantly higher intrinsic disorder in average (41.1 ± 6.4%) compared to those in a prokaryotic proteome (15.6 ± 6.5%), consistent with previous studies [14, 28]. This trend stands for the average disorder contents of all residues from the proteomes (47.5 ± 6.4% for eukaryotes compared to 32.9 ± 1.4% for prokaryotes). Proteomes from the archaeon *N. equitans* and bacterium *Rickettsiales* have the lowest disorder content at the protein level (7.0% for *N. equitans* and 7.7% for *Rickettsiales*) for the systems examined. As the smallest known archaeon, *N. equitans* is an obligate symbiont on the other archaeon *I. hospitalis*, which is the smallest known free-living archaeon [29]. The free-living alphaproteobacterium *Rickettsiales*, on the other hand, was suggested to be a living candidate that is close to the ancient endosymbiotic alphaproteobacteria that were merged into an archaeon and eventually transferred into the mitochondria of the first eukaryotic cell [30]. These two symbiotic or presymbiotic organisms have retained more ordered proteins compared to other free-living bacteria and Archaea surveyed here.

Consistent with previous studies [15], the proteins from the mitochondrion (88,405 proteins from 6119 species) and chloroplast (80,807 proteins form 935 species) sets have relatively low disorder contents compared to the proteins encoded in nuclear genes of eukaryotic organisms, for example, the mitochondrial protein set has a considerably lower disorder content of 8.6% at protein level. The mitochondria have lost most of their ancestral genes either by transferring to the nucleus or by being discarded [31]. Here, we show that the mitochondrial proteins have relatively low disorder contents (i.e., highly ordered) at both the protein and the residue levels (Table 1). The genes retained in the mitochondrial genomes have been proposed preferentially to encode core proteins involved in electron transfers [32], and a colocalization of the redox regulation (CoRR) mechanism was proposed to explain why the mitochondrial and chloroplastic organelles retain their own genes, or proteins [33, 34]. Our analysis indicates that the chloroplast genes have their proteins with disorder contents close to the free-living prokaryotes, but higher than those from the symbiotic Archaeon *N. equitans* and alphaproteobacterium *Rickettsiales*, as well as the mitochondrial set (Table 1).

The proteomes of two giant DNA viruses (giruses), the Mimivirus and Pandoravirus, were also analyzed. The numbers of proteins encoded in these two giruses are comparable to the prokaryotic proteomes. The disorder content of the proteome of the Mimivirus is larger than that of the prokaryotes, but smaller than that of the eukaryotes surveyed here. However, the Pandoravirus has a proteome with disorder content close to that of the eukaryotes.

Finally, the viral and plasmid gene sets were analyzed. The viral gene set contains 237,463 genes collected from 4942 strains and the plasmid set contains 95,214 genes cultivated from 985 bacteria. Interestingly, the proteins from these two sets yield similar trends in both length and disorder distributions.

### 2.2. Definition of the LD Space

**Figure 2:**
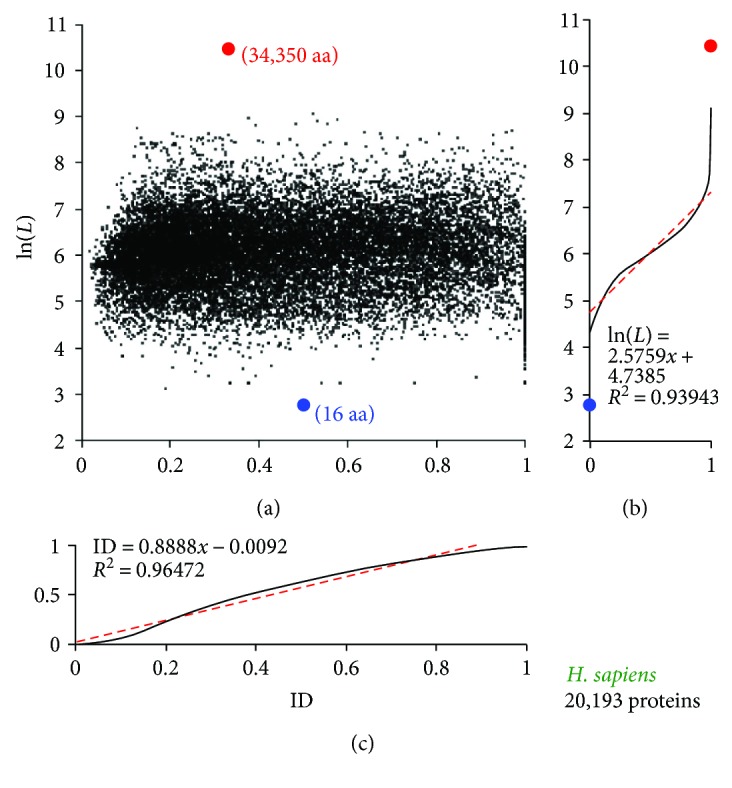
Protein distributions for the human (*H. sapiens*) proteome in the LD space defined by ln(*L*) (the protein length in a logarithm scale) and ID (protein intrinsic disorder contents with 1.0 corresponding to proteins with 100% residues disordered and 0.0 corresponding to proteins with 0% residues disordered). The distributions in the hierarchical scale are shown in (b) and (c), respectively (see text). Linear fittings of ln(*L*) and ID are shown in red dashed lines with satisfactory *R* ^2^ and hence support the linear participations shown in Table 2. The blue and red dots indicate the shortest (16 aa) and longest (34,350 aa) proteins, respectively.

Consistent with a previous report [3], exponential distributions of the protein lengths (*L*) in all proteomes and protein data sets have been observed. In this analysis, all proteins of a proteome or protein set have been ranked hierarchically from the shortest to the longest, and the proteins then distribute linearly on ln(*L*) (the natural base was used for the logarithm function in this study). Similar linear distribution trend is observed for the percentage of residues located in the IDPR, ID (Figure 2). Therefore, a two-dimensional LD space could be defined with one phase for the content of the protein intrinsic disorder, ID, and the other phase for the logarithm of the protein length, ln(*L*). Figure 2 exemplifies the protein distribution in the LD space of the human proteome.

### 2.3. Dependency of the Two Attributes for the LD Space

**Figure 3:**
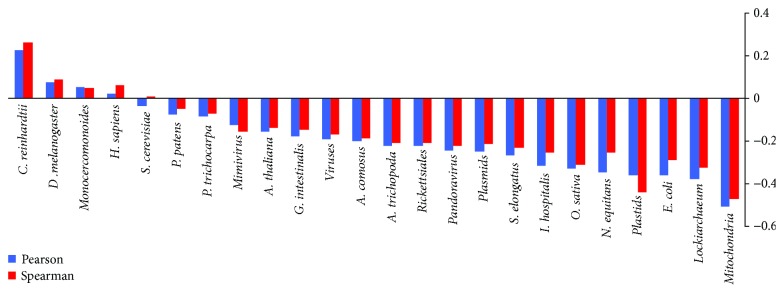
Pearson's (blue) and Spearman's (red) correlation coefficients (CCs) between ln(*L*) and ID of the proteins in proteomes and gene sets surveyed in the present work. All species were ranked by the Pearson's CCs from the highest positive (*C. reinhardtii*) to highest negative (mitochondrial gene set).

We defined a two-dimensional LD space with the two attributes, ln(*L*) and ID, and these two attributes need to be independent of each other. Therefore, we calculated the correlation coefficients (CCs) between ln(*L*) and ID of proteins in all proteomes and protein sets (Figure 3). Pearson's and Spearman's CCs for all proteins (811,600 entries, Table 1 and S1) are −0.101 and −0.129, respectively. The overall slight negative CC (anticorrelation) indicates that there may be a trend that shorter proteins have averagely higher disorder contents than the longer proteins. However, the anticorrelational trend does not hold for all species surveyed in this study and positive CC values were found, too, such as in the animals (human and fruit fly) and green algae *C. reinhardtii* (Table S1). The variations in the correlational trends between ln(*L*) and ID, therefore, may have been driven by the evolutionary processes rather than a cause-and-effect relationship. As such, the validity of the protein LD space and the related architecture of protein distributions in the LD space (i.e., the “fingerprint”) should be discussed in an evolutionary framework (see below).

### 2.4. Architecture of Protein Distribution (Fingerprint) in the LD Space

The most thoroughly annotated animal and plant genomes may be those of human (*H. sapiens*) and *Arabidopsis thaliana*, respectively. Using proteomes form the two representative animal and plant, the protein distributions of proteomes in the LD space were converted to the protein-density contour maps in Figure 1(a) (see Materials and Methods). As we will show below, this approach may be useful in comparative proteomes/genomics.

**Figure 4:**
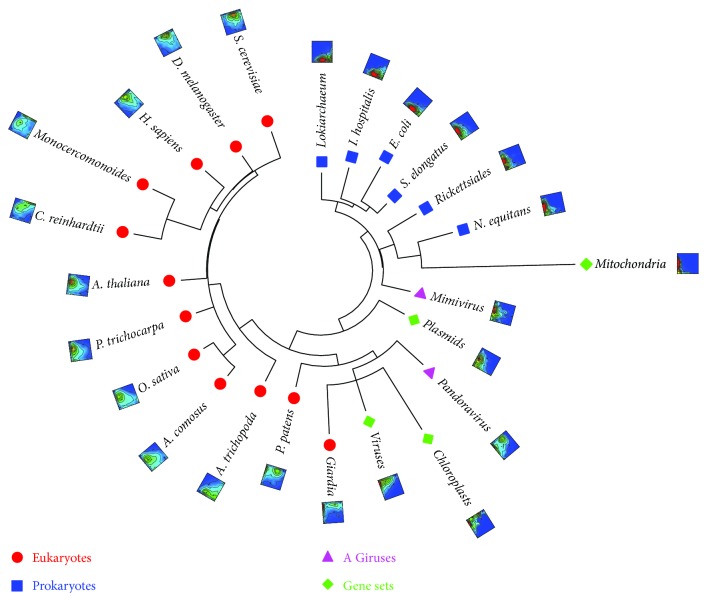
The phylogenetic tree reconstructed using the protein distribution densities on the LD space. The protein density distributions in the LD space for each species or gene set are also shown (Figure S1 in Supplementary Materials shows higher resolution figures). MEGA5 [69] was used to plot the tree.

At a first glance, the plant proteome has more proteins of medium lengths (~5.7 < ln(*L*) < 6.4 or ~300 < *L* < 600) and relatively low disorder contents (ID < 0.3) whereas the animal proteome contains more long and disordered proteins (e.g., *L* > 600 and ID > 0.5). This may partly explain the slightly positive correlations between ln(*L*) and ID in the animal proteomes but negative correlations in the plant proteomes. The protein distribution contour maps of other proteomes and gene sets can be found in Figure S1 in Supplementary Materials and have been trimmed in the phylogenetic tree in Figure 4 (see below).

It is straightforward to visualize the differences of these two proteomes using the differential contour in Figure 1(b). The *H. sapiens* proteome has 657 short proteins (i.e., *L* < 100 or ln(*L*) < 4.6), among which 294 (1.5% of all proteins) are considered disordered (ID > 0.5); in the *A. thaliana* proteome, 888 (3.2%) out of 2292 short proteins are disordered. On the other hand, in the *H. sapiens* proteome, 1135 (5.6%) out of 2384 long proteins (i.e., *L* ≥ 1000 or ln(*L*) ≥ 6.9) are disordered; whereas, in the *A. thaliana* proteome, 306 (1.1%) out of 1157 long proteins are disordered. Therefore, a significant difference between the animal (*H. sapiens*) and the plant (*A. thaliana*) could be recognized as that the former has more long disordered proteins, whereas the latter has more short disordered proteins. This difference shown in Figure 1(b) allows us to narrow down the protein/gene distributions related to the architectural differences between the two organisms.

A recent report also indicates that the overall disorder contents of the *A. thaliana* proteome are lower than those of the *H. sapiens* proteome [35], which was attributed that more IDP genes functioning in environmental adaptations may have been enriched in plants [35]. Based on our analysis and the apparent abundance of the short disordered proteins in *A. thaliana* compared to *H. sapiens* (Figure 1(b)), we focus on the 888 short (<100 aa, see above) IDP (sIDP) genes of *A. thaliana*. Among these genes, the GO annotations of 203 sIDPs could not be identified, that is, they may be considered among the “dark matter” of the *A. thaliana* proteome [36]. However, among the 685 annotated sIDPs (occupying 545 GO terms), only 20 (~0.2% of all sIDPs) with 32 GO annotations were included in the previous analysis showing “enrichment” of 74 GO annotations related to the environmental adaptations in *A. thaliana* compared to *H. sapiens* [35]. Based on our analysis, this enrichment might not be significant for the sIDPs. We suggest that it might be possible that in animals and other organism (e.g., the green algae *C. reinhardtii*), some of the sIDPs had been lost whereas long IDPs were enriched. Here, GO annotations of the plant genes were adopted from the plant comparative genomics database PLAZA 3.0 [37].

### 2.5. Phylogeny Reconstructed Based on Protein Distribution Densities in the LD Space

As the first test concerning whether our classification of proteomes and protein sets is biologically reasonable, we generated phylogenetic trees based on the protein distribution densities in the fingerprints of proteomes without performing any protein sequence comparison and alignments. Here, aiming to quantify the *architectural* differences among proteomes, the LD space was divided into *M* × *N* blocks and then, the distance between two species A and B was calculated using a Euclidian-type formula based on the protein distributions in all blocks (see (3) in Materials and Methods). In this *architectural-*distance calculation, no rigorous biological function annotations and/or genomic comparisons using BLAST or other protocols are required.

**Figure 5:**
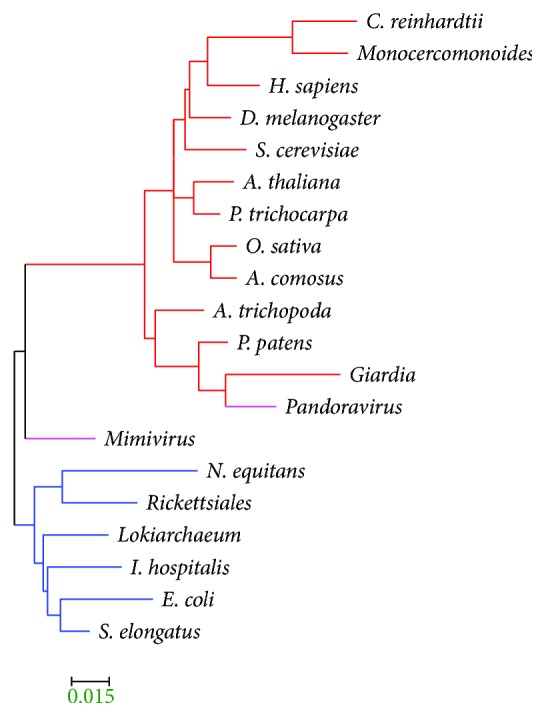
The phylogenetic tree reconstructed from the protein distributions in the LD space using *M* = *N* = 10 in (3) in Materials and Methods. Eukaryotes are in red, prokaryotes (bacteria and Archaea) in blue and giruses in pink branches. MEGA5 [69] was used to plot the trees. Because this tree is based on normalized protein densities (of 100 blocks in the *M* = *N* = 10 tree here), the branch length of the tree is relatively small with a scale bar of 0.015.

By dividing the LD space with *M* = *N* = 10 (Table 2), the distance matrices for all proteomes including those from giruses (Table 1) were calculated and converted to phylogenetic trees as shown in Figure 5. We also tested the 5 × 5 or 2 × 2 partitioning; the 10 × 10 partitioning of the LD space seems to yield relatively high accuracy (Table S2 and Figure S2 in Supplementary Materials). Nevertheless, some of the key properties are not very sensitive to the *M* and *N* values. Several interesting features have been found in the trees that we reconstructed: (1) the eukaryotes are clearly separated from the prokaryotes and (2) plants and animals are grouped together, even the eudicot plants (*A. thaliana* and *P. trichocarpa*) and monocot plants (*O. sativa* and *A. comosus*) are separated. The tree in Figure 5 correctly puts *A. trichopoda* before the other plant species and after *P. patents*. Interestingly, it is consistent with our understanding of the plants-fungi-animals phylogenetic relationships [38] and stays in the framework of the natural classification of three domains of life [39]. Based on the phylogenetic tree, the definition of the protein LD space might be considered meaningful to the proteomes, at least to those chosen in present work.

## 3. Discussion

To the best of our knowledge, this is the first time to classify proteomes and protein sets based on the protein distribution densities in the LD space (fingerprints), and a detailed comparison with the previous work is therefore not straightforward. Nevertheless, the survey of protein distributions in terms of each of the two attributes is consistent with the work published previously. We noticed that the eukaryotic proteomes do not always exhibit averagely longer proteins than the prokaryotic proteomes. Our observation on protein disorder agrees well with the previous finding, that is, the average disorder contents in eukaryotic proteins are indeed higher than those in prokaryotic proteins. We have also generated phylogenetic trees based on the protein distribution densities in the fingerprints of proteomes, and this allows us to make some comparisons of the results that we obtained here with the knowledge in the field and to examine the consistency and differences with earlier investigations. Such comparison may also provide certain alternative views that were generated through this unique approach.

### 3.1. Giant DNA Viruses and the Tree of Life

It has been in the debate over the years concerning if viruses should be included in the tree of life [40, 41] or if they are alive at all [42, 43]. The discovery of Mimivirus [44] that belongs to the nucleocytoplasmic large DNA viruses (NCLDV) and the following discoveries of other giant DNA viruses (giruses) [45], for example, the Pandoravirus with a genome size exceeding some of the cellular organisms [46], invoked questions on if a “fourth domain” should be added to the tree of life [46, 47] and potentially important roles that viruses played in eukaryogenesis [48]. Interestingly, we found that Mimivirus is located in between the Eukaryota and prokaryote (Archaea + Eubacteria) branches, that is, at the prokaryote-to-eukaryote transition zone. This is consistent with the original phylogenetic analysis inferred based on seven universally conserved protein sequences [44]. The Pandoravirus, on the other hand, is located within the Eukaryota branch. The vast majority (>93%) of the Pandoravirus genes exhibit no homology to anything known [46]; however, our approach puts it in the same branch of the parasite Giardia (Figure 5(c)), owing to the abundance of short proteins (both in ordered and disordered states) in these two organisms (Figure S1).

### 3.2. Organelles

The phylogenetic tree with the viral and organelle (mitochondria, chloroplasts, and plasmids) gene sets is shown in Figure 4 along with the fingerprints in the LD space. In this tree, the viral gene set is located in the same branch as the Pandoravirus. The plasmid gene set is located in between prokaryotic and eukaryotic branches, or more accurately, between Mimivirus and Pandoravirus. These results suggest the importance of horizontal gene transfers in eukaryogenesis carried by the viral and plasmid genes.

**Figure 6:**
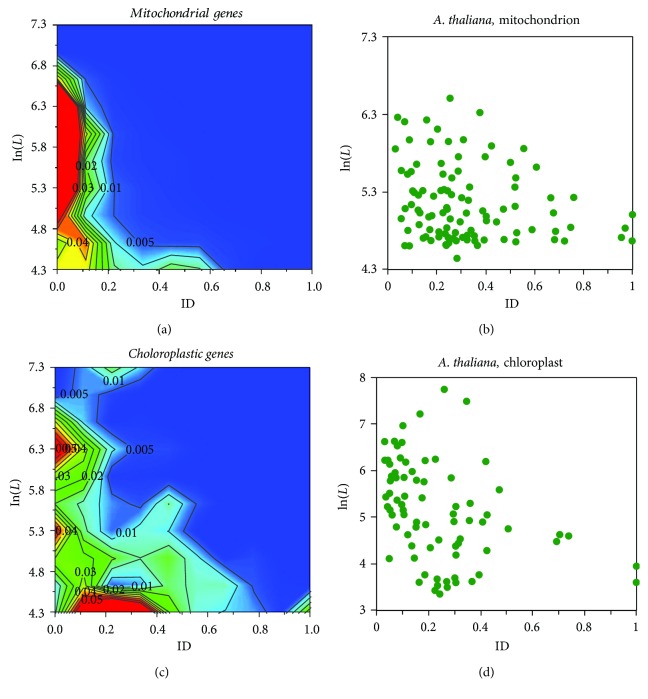
Protein distributions in the LD space for (a) the mitochondrial gene set, (b) the mitochondrial genes in *A. thaliana*, (c) the chloroplast gene set, and (d) the chloroplastic genes in *A. thaliana*.

In Figure 4, the mitochondrial gene set sits in the same branch as the symbiont *N. equitans* and alphaproteobacterium *Rickettsiales*, owing to that majorities of the proteins in these proteomes and protein set are highly ordered (Table 1). The chloroplast set is located at the same branch as the viral gene set and *Giardia* (Figure 4). Using the full set of annotated mitochondrial genomes for 2015 species, a recent report [32] revealed that the proteins retained in the eukaryotic mitochondria are preferentially the structural cores in the electron transportation chains. Our survey with the mitochondrial proteins obtained from the NCBI database indicates that the mitochondrial proteins are mainly structurally ordered (Figure 6(a)), thereby possibly structurally and functionally conserved, too. However, using the model plant species *A. thaliana* as an example, the mitochondrial protein distribution in the LD space (Figure 6(b)) does not match that from the mitochondrial gene set (Figure 6(a)). This inconsistency may originate from a considerable amount of highly disordered proteins retained in the mitochondria. For instance, *A. thaliana* has 115 mitochondrial genes, 23 of which are IDPs (i.e., ID ≥ 0.5; here, ID refers to the ratio of residues). However, we found that 19 (out of 23) mitochondrial IDPs have unknown functions involved in unknown biological processes (Table S3 in Supplementary Materials), immediately raising a question on the validity of the results obtained from annotated mitochondrial genomes (Figure 6(a) in the present study and [32]). The protein distribution profile of *A. thaliana* chloroplast (Figure 6(d)) resembles that of the collective chloroplast gene set (Figure 6(c)). Only 6 out of 85 *A. thaliana* proteins are IDPs, all of which have been annotated as ribosomal proteins (Table S3).

## 4. Conclusion

Our two-dimensional contour maps (or proteome fingerprints) based on the protein distribution densities in the LD space show distinct patterns for different organisms and protein sets and may therefore be used for classification of proteomes and protein sets. The phylogenetic trees generated based on the protein distribution densities from the fingerprints were found to be meaningful, as they seem to contain important information of evolution. Thus, the proposed approach and its further extension may represent a useful and alternative way for proteome classification and comparison. It should be pointed out that although in the present work we used protein lengths (*L*) and protein intrinsic disorder contents (D) as the basic attributes, other attributes (not limited to those from proteins) may be introduced as well. One can imagine that one of the properties for the attributes would be that protein distributions in terms of the new attributes would be different for different proteomes (protein sets) so that the purpose of classification of proteomes (protein sets) can be achieved.

## 5. Materials and Methods

### 5.1. Proteomes and Gene Set

The plant proteomes in this study were downloaded from Phytozome, and the proteomes of bacteria, Archaea, and animals were downloaded from UniProt; the organelle protein sets were obtained from NCBI, at or before December 2016.

Here, we surveyed 12 eukaryotic proteomes from two animal species *Homo sapiens* [49, 50] and *Drosophila melanogaster* [51], two monocot plant species *Oryza sativa L. ssp. indica* [52] and *Ananas comosus* [53], two dicot plant species *Arabidopsis thaliana* [54] and *Populus trichocarpa* [55], the basal angiosperm *Amborella trichopoda* [56], the moss *Physcomitrella patens* [57], the fungus *Saccharomyces cerevisiae strain S288C* [58], the green algae *Chlamydomonas reinhardtii* [59], the metamonada Giardia (previously known as an Archezoa that lacks conventional mitochondrion) [60], and *Monocercomonoides sp. PA203* that completely lacks the mitochondrial or mitochondrial-derived genes [61]. We also analyzed three bacterial species *Escherichia coli K12 MG1655* [62], the cyanobacterium *Synechococcus elongatus PCC 7942* [63], and the alphaproteobacterium *Rickettsiales bacterium Ac37b* [64] and three Archaea species *Ignicoccus hospitalis kin4/i*, *Nanoarchaeum equitans* [29], and *Lokiarchaeum sp. GC14_75* [65]. Two giant DNA-viruses (giruses) were also analyzed, including the *Mimivirus* [44] and *Pandoravirus salinus* [46]. In addition, we downloaded several gene collections from the NCBI gene libraries containing the viral set (237,463 genes), plasmid set (95,214 genes), mitochondrial set (88,405 genes), and chloroplast set (80,807 genes). Table 1 gives a summary of the proteomes and gene sets.

The proteomes and gene sets listed above comprise 811,600 proteins, among which 2401 proteins (~0.3%) contain unknown “*X*” residues and were excluded for analysis in this work.

It should be pointed out that in the present analysis, only the primary protein at each gene locus is selected. The poplar (*P. trichocarpa*) proteome [55] was selected to test the potential influence of the versions of the proteomes and splicing alternatives. From the *P. trichocarpa* genome, there are three versions (v01, v02, and v03) of the proteomes, of which the v03 proteome has 41,434 primary proteins and 31,579 splicing alternatives (73,013 proteins in total). Using the primary proteins of all three versions and the full proteome of the v03 version as separated entries, a phylogenetic tree was constructed (Figure S3 in Supplementary Materials) and there is little difference with or without using alternative splicing proteins or by using different proteome versions.

### 5.2. Intrinsic Disorder (ID) Prediction

The PONDR-VSL2 algorithm [66] was applied to predict the ID content of all residues in a protein. This program had achieved ~81% accuracy for both short and long proteins. By default, a residue is in an ordered state if its PONDR score is less than 0.5, but in a disordered state when the PONDR score is larger than or equal to 0.5. PONDR scores of 0 and 1 corresponding to the fully ordered and fully disordered states, respectively. Here, this criterion was adopted and extended to calculate the ID content of a protein:

(1) $$\begin{array}{c} {ID}_{pep}=\frac{N_{D}}{L}, \end{array}$$

where *N*_D_ is the number of disordered residues and *L* is the total number of residues of the protein (i.e., protein length). ID_pep_ is also termed as the “*rough definition*” of the disorder contents in [27] and ranges from 0 to 1, with 0 and 1 corresponding to the fully ordered and fully disordered proteins, respectively.

It had been suggested that the total proteome information content (PIC) could be defined as the total number of amino acids of the primary proteins (longest isoform at each gene locus) that the proteome carries [67]. In accordance with this definition, we also calculated the average intrinsic disorder content per residue as

(2) $$\begin{array}{c} {ID}_{res}=\overset{X}{\underset{i=1}{\sum }}\frac{D_{i}}{X}, \end{array}$$

where *Χ* is the total number of amino acids and *D_i_* is the PONDR score of the *i*th residue of the proteome or protein set. ID_res_ corresponds to the definition adapted in [27]. Both ID_pep_ and ID_res_ are listed in Table 1. Because in present work distributions of genes (or proteins) are used to discuss the evolutionary dynamics, ID_pep_ (simplified as ID in the main text) had been chosen to act as one of the attributes of the LD space.

### 5.3. Generation of the Fingerprints and Phylogenetic Analysis

To generate the fingerprints, the LD space of species *X* was first divided into *M* × *N* blocks (e.g., Table 2), *M* for ln(*L*) and *N* for ID. This separation is reasonable because both ln(*L*) and ID exhibit linearity (Figure 2). Then, the protein density in the *ij*th block (*i* in ln(*L*) and *j* in ID%) is calculated as *X*_*ij*_ = *n*_*ij*_/*n*_tot_, where *n_ij_* is the number of proteins in the *ij*th block and *n*_tot_ = ∑_*l*=1_^*M*^∑_*d*=1_^*N*^ *n*_*ld*_ is the total number of proteins in the proteome of species *X*. Normalization of the protein density is realized by default since ∑*X*_*ij*_ = 1.

Using the protein densities, the distance between two organisms A and B can be calculated using the Euclidean equation:

(3) $$\begin{array}{c} r_{AB}=\sqrt{\overset{M}{\underset{l=1}{\sum }}\overset{N}{\underset{d=1}{\sum }}{\left(A_{ld}-B_{ld}\right)}^{2},} \end{array}$$

where *r_AB_* is the distance between A and B and *X_ij_* (*X* = *A* or *B*) is the protein density in the *ij*th block. The calculated distance matrix is converted to the phylogenetic tree using the neighbor-joining method by the T-REX web server [68]. *M* and *N* and detailed block separations may serve as variables to fine tune the final phylogenetic tree. As a proof of concept, the reconstructed phylogenetic tree using *M* = *N* = 10 is shown in Figure 5.

The overall working flow of phylogenetic tree reconstruction is as follows: selection of the proteomes and protein sets → calculations and statistics of the intrinsic disorder contents (ID) and protein length of primary proteins (logarithm, ln(*L*)) → calculations of the protein densities in all blocks (Table 2) → calculations of the Euclidian distance between each pair of proteomes or protein sets (3) → reconstruction of the phylogenetic tree based on the distance matrix.

## Figures and Tables

**Table 1:** A summary of the proteomes and gene sets.

Domain^a^	Species	Gene number^b^	Ave^c^	Med^c^	Max^c^	Min^c^	ID_pep_%^b,d^	ID_res_%^b,e^
Eukaryota	*H. sapiens*	20,193	561.0	417	34,350	16	45.2	49.3
	*D. melanogaster*	13,700	537.2	396	22,949	11	44.3	49.0
	*S. cerevisiae*	5917	494.1	405	4910	16	38.1	44.6
	*A. thaliana*	27,407	405.2	348	5393	7	36.8	43.6
	*P. trichocarpa*	41,434	385.0	317	5410	29	35.5	42.6
	*A. comosus*	29,772	372.6	288	5407	31	39.5	45.4
	*O. sativa*	48,788	376.1	290	4957	5	38.0	44.5
	*A. trichopoda*	26,460	317.0	218	4990	29	37.5	43.9
	*C. reinhardtii*	17,819	732.9	498	23,859	31	54.8	61.9
	*P. patens*	32,400	351.9	250	5199	13	40.2	45.5
	*G. intestinalis*	9667	353.8	147	8161	33	35.1	41.7
	*Monocercomonoides*	16,780	784.6	393	14,902	49	52.7	60.1

Archaea	*Lokiarchaeum*	5348	268.4	224	3592	20	20.0	33.0
	*I. hospitalis*	1434	278.3	240	1392	33	20.4	34.3
	*N. equitans*	540	280.2	228	2197	45	7.0	30.6

Bacteria	*E. coli*	4140	316.9	282	2358	14	17.5	32.2
	*S. elongatus*	2612	305.3	258	1807	29	20.8	34.3
	*Rickettsiales*	1780	365.2	251	2243	31	7.7	32.8

Giruses	*Mimivirus*	979	356.7	289	2959	25	25.0	36.6
	*Pandoravirus*	2541	259.2	178	2321	26	36.4	43.5

Gene sets	*Viruses*	237,463	251.8	154	8573	9	28.0	38.8
	*Plasmids*	95,214	258.9	206	16,990	9	27.2	38.1
	*Mitochondria*	88,405	286.1	261	2640	13	8.6	20.0
	*Plastids*	80,807	280.0	156	5242	12	20.5	32.0

All proteins^f^		811,600	325.7	225	34,350	5	32.2	39.8

^a^Proteomes in the three domains of life; the giant DNA viruses (giruses) and collective protein sets are listed after the cellular species; ^b^Total gene numbers; ^c^Protein length statistics: Ave: average; Med: median; Max: maximal; Min: minimal protein lengths; ^d^Percentage of the intrinsically disordered proteins in the proteome or gene set; ^e^Average intrinsic disorder contents of all residues carried by the proteome or gene set; ^f^All proteins studied in the present work. The protein length statistics covers all proteins in a proteome or gene set; however, the proteins with unknown sequence(s) (*X* residues) are excluded in the intrinsic disorder calculations.

**Table 2:** Intervals that partition the LD spaces into *M* × *N* blocks with *M* = *N* = 10.

	Number	1	2	3	4	5	6	7	8	9	10
10 × 10	ln(*L*)	(0,4.6)	(4.6,4.9)	(4.9,5.2)	(5.2,5.5)	(5.5,5.8)	(5.8,6.1)	(6.1,6.4)	(6.4,6.7)	(6.7,7.0)	(7.0,∞)
	*L*	(1100)	(101,135)	(135,182)	(182,245)	(245,331)	(331,446)	(446,602)	(602,813)	(813,1097)	(1097,∞)
	ID%	(0,0.1)	(0.1,0.2)	(0.2,0.3)	(0.3,0.4)	(0.4,0.5)	(0.5,0.6)	(0.6,0.7)	(0.7,0.8)	(0.8,0.9)	(0.9,1.0)

## Supplementary Materials

Supplementary Materials Table S1. Correlation coefficients between ln(*L*) and ID%. Table S2. Intervals that partition the LD spaces into *M* × *N* blocks with *M* = *N* = 2 and 5. Table S3. IDPs in the mitochondrion and chloroplast of *A. thaliana*. Figure S1. Protein-density contour maps (see Figure 1(a) in main text for the scale bar). Figure S2. Phylogenetic trees reconstructed from the protein distributions in the LD space using A—(*M* = *N* = 2) and B (*M* = *N* = 5). Eukaryotes are in red, prokaryotes (bacteria and Archaea) in blue, and giruses in pink branches. MEGA5 (1) was used to plot the trees. Compared to that of the *M* = *N* = 10 tree (Figure 5), the branch length of the *M* = *N* = 10 tree is larger. Figure S3. Phylogenetic tree reconstructed from gene densities on the LD space. Different versions (v01–v03) of the *P. trichocarpa* proteomes have been used. By default of the present work, only proteins from primary transcripts are chosen for all proteomes. Here, for *P. trichocarpa* proteome v03, we tested both the primary transcripts (41,434 proteins) and all transcripts (73,013 proteins). We show here that progressive improvements including the splicing variants did not make significant changes in the phylogeny.

## References

[1] Thirumalai D., O'Brien E. P., Morrison G., Hyeon C. (2010). Theoretical perspectives on protein folding. *Annual Review of Biophysics*.

[2] Dill K. A., Ghosh K., Schmit J. D. (2011). Physical limits of cells and proteomes. *Proceedings of the National Academy of Sciences of the United States of America*.

[3] Zhang J. Z. (2000). Protein-length distributions for the three domains of life. *Trends in Genetics*.

[4] Brocchieri L., Karlin S. (2005). Protein length in eukaryotic and prokaryotic proteomes. *Nucleic Acids Research*.

[5] Uversky V. N. (2002). Natively unfolded proteins: a point where biology waits for physics. *Protein Science*.

[6] Uversky V. N., Dunker A. K. (2010). Understanding protein non-folding. *Biochimica et Biophysica Acta (BBA) - Proteins and Proteomics*.

[7] Tompa P. (2012). Intrinsically disordered proteins: a 10-year recap. *Trends in Biochemical Sciences*.

[8] Uversky V. N. (2013). A decade and a half of protein intrinsic disorder: Biology still waits for physics. *Protein Science*.

[9] Berlow R. B., Dyson H. J., Wright P. E. (2015). Functional advantages of dynamic protein disorder. *FEBS Letters*.

[10] Dunker A. K., Bondos S. E., Huang F., Oldfield C. J. (2015). Intrinsically disordered proteins and multicellular organisms. *Seminars in Cell & Developmental Biology*.

[11] Uversky V. N. (2015). The multifaceted roles of intrinsic disorder in protein complexes. *FEBS Letters*.

[12] Tompa P., Schad E., Tantos A., Kalmar L. (2015). Intrinsically disordered proteins: emerging interaction specialists. *Current Opinion in Structural Biology*.

[13] Wright P. E., Dyson H. J. (2015). Intrinsically disordered proteins in cellular signalling and regulation. *Nature Reviews Molecular Cell Biology*.

[14] Xue B., Dunker A. K., Uversky V. N. (2012). Orderly order in protein intrinsic disorder distribution: disorder in 3500 proteomes from viruses and the three domains of life. *Journal of Biomolecular Structure and Dynamics*.

[15] Yruela I., Contreras-Moreira B. (2012). Protein disorder in plants: a view from the chloroplast. *BMC Plant Biology*.

[16] Uversky V. N., Dave V., Iakoucheva L. M. (2014). Pathological unfoldomics of uncontrolled chaos: intrinsically disordered proteins and human diseases. *Chemical Reviews*.

[17] Iakoucheva L. M., Brown C. J., Lawson J. D., Obradović Z., Dunker A. K. (2002). Intrinsic disorder in cell-signaling and cancer-associated proteins. *Journal of Molecular Biology*.

[18] Joerger A. C., Fersht A. R. (2016). The p53 pathway: origins, inactivation in cancer, and emerging therapeutic approaches. *Annual Review of Biochemistry*.

[19] Uversky V. N., Na I., Landau K. S., Schenck R. O. (2017). Highly disordered proteins in prostate cancer. *Current Protein & Peptide Science*.

[20] Uversky V. N. (2010). Targeting intrinsically disordered proteins in neurodegenerative and protein dysfunction diseases: another illustration of the D^2^ concept. *Expert Review of Proteomics*.

[21] Metallo S. J. (2010). Intrinsically disordered proteins are potential drug targets. *Current Opinion in Chemical Biology*.

[22] Marasco D., Scognamiglio P. L. (2015). Identification of inhibitors of biological interactions involving intrinsically disordered proteins. *International Journal of Molecular Sciences*.

[23] Lazo J. S., Sharlow E. R. (2016). Drugging undruggable molecular cancer targets. *Annual Review of Pharmacology and Toxicology*.

[24] Kumar D., Sharma N., Giri R. (2017). Therapeutic interventions of cancers using intrinsically disordered proteins as drug targets: c-Myc as model system. *Cancer Informatics*.

[25] Ambadipudi S., Zweckstetter M. (2016). Targeting intrinsically disordered proteins in rational drug discovery. *Expert Opinion on Drug Discovery*.

[26] Midic U., Oldfield C. J., Dunker A. K., Obradovic Z., Uversky V. N. (2009). Protein disorder in the human diseasome: unfoldomics of human genetic diseases. *BMC Genomics*.

[27] Lobanov M. Y., Galzitskaya O. V. (2015). How common is disorder? Occurrence of disordered residues in four domains of life. *International Journal of Molecular Sciences*.

[28] Peng Z., Yan J., Fan X. (2015). Exceptionally abundant exceptions: comprehensive characterization of intrinsic disorder in all domains of life. *Cellular and Molecular Life Sciences*.

[29] Podar M., Anderson I., Makarova K. S. (2008). A genomic analysis of the archaeal system Ignicoccus hospitalis-Nanoarchaeum equitans. *Genome Biology*.

[30] Ball S. G., Bhattacharya D., Weber A. P. (2016). Pathogen to powerhouse. *Science*.

[31] Neupert W. (2016). Mitochondrial gene expression: a playground of evolutionary tinkering. *Annual Review of Biochemistry*.

[32] Johnston I. G., Williams B. P. (2016). Evolutionary inference across eukaryotes identifies specific pressures favoring mitochondrial gene retention. *Cell Systems*.

[33] Allen J. F. (2015). Why chloroplasts and mitochondria retain their own genomes and genetic systems: colocation for redox regulation of gene expression. *Proceedings of the National Academy of Sciences of the United States of America*.

[34] Allen J. F., de Paula W. B. M., Puthiyaveetil S., Nield J. (2011). A structural phylogenetic map for chloroplast photosynthesis. *Trends in Plant Science*.

[35] Pietrosemoli N., Garcia-Martin J. A., Solano R., Pazos F. (2013). Genome-wide analysis of protein disorder in *Arabidopsis thaliana*: implications for plant environmental adaptation. *PLoS One*.

[36] Perdigao N., Heinrich J., Stolte C. (2015). Unexpected features of the dark proteome. *Proceedings of the National Academy of Sciences of the United States of America*.

[37] Proost S., Van Bel M., Vaneechoutte D. (2015). PLAZA 3.0: an access point for plant comparative genomics. *Nucleic Acids Research*.

[38] Wainright P. O., Hinkle G., Sogin M. L., Stickel S. K. (1993). Monophyletic origins of the metazoa: an evolutionary link with fungi. *Science*.

[39] Woese C. R., Kandler O., Wheelis M. L. (1990). Towards a natural system of organisms: proposal for the domains Archaea, bacteria, and Eucarya. *Proceedings of the National Academy of Sciences of the United States of America*.

[40] Moreira D., Lopez-Garcia P. (2009). Ten reasons to exclude viruses from the tree of life. *Nature Reviews Microbiology*.

[41] Claverie J.-M., Ogata H. (2009). Ten good reasons not to exclude giruses from the evolutionary picture. *Nature Reviews Microbiology*.

[42] Koonin E. V., Starokadomskyy P. (2016). Are viruses alive? The replicator paradigm sheds decisive light on an old but misguided question. *Studies in History and Philosophy of Science Part C: Studies in History and Philosophy of Biological and Biomedical Sciences*.

[43] Forterre P. (2016). To be or not to be alive: how recent discoveries challenge the traditional definitions of viruses and life. *Studies in History and Philosophy of Science Part C: Studies in History and Philosophy of Biological and Biomedical Sciences*.

[44] Raoult D., Audic S., Robert C. (2004). The 1.2-megabase genome sequence of Mimivirus. *Science*.

[45] Fischer M. G. (2016). Giant viruses come of age. *Current Opinion in Microbiology*.

[46] Philippe N., Legendre M., Doutre G. (2013). Pandoraviruses: amoeba viruses with genomes up to 2.5 Mb reaching that of parasitic eukaryotes. *Science*.

[47] Moreira D., Lopez-Garcia P. (2015). Evolution of viruses and cells: do we need a fourth domain of life to explain the origin of eukaryotes?. *Philosophical Transactions of the Royal Society B-Biological Sciences*.

[48] Forterre P., Gaia M. (2016). Giant viruses and the origin of modern eukaryotes. *Current Opinion in Microbiology*.

[49] Venter J. C., Adams M. D., Myers E. W. (2001). The sequence of the human genome. *Science*.

[50] Olivier M., Aggarwal A., Allen J. (2001). A high-resolution radiation hybrid map of the human genome draft sequence. *Science*.

[51] Adams M. D., Celniker S. E., Holt R. A. (2000). The genome sequence of *Drosophila melanogaster*. *Science*.

[52] Yu J., Hu S., Wang J. (2002). A draft sequence of the rice genome (*Oryza sativa* L. ssp. *indica*). *Science*.

[53] Ming R., VanBuren R., Wai C. M. (2015). The pineapple genome and the evolution of CAM photosynthesis. *Nature Genetics*.

[54] I. Arabidopsis Genome (2000). Analysis of the genome sequence of the flowering plant *Arabidopsis thaliana*. *Nature*.

[55] Tuskan G. A., DiFazio S., Jansson S. (2006). The genome of black cottonwood, *Populus trichocarpa* (Torr. & Gray). *Science*.

[56] P. Amborella Genome (2013). The *Amborella* genome and the evolution of flowering plants. *Science*.

[57] Rensing S. A., Lang D., Zimmer A. D. (2008). The *Physcomitrella* genome reveals evolutionary insights into the conquest of land by plants. *Science*.

[58] Cherry J. M., Hong E. L., Amundsen C. (2012). Saccharomyces genome database: the genomics resource of budding yeast. *Nucleic Acids Research*.

[59] Merchant S. S., Prochnik S. E., Vallon O. (2007). The *Chlamydomonas* genome reveals the evolution of key animal and plant functions. *Science*.

[60] Aurrecoechea C., Brestelli J., Brunk B. P. (2009). GiardiaDB and TrichDB: integrated genomic resources for the eukaryotic protist pathogens *Giardia lamblia* and *Trichomonas vaginalis*. *Nucleic Acids Research*.

[61] Karnkowska A., Vacek V., Zubacova Z. (2016). A eukaryote without a mitochondrial organelle. *Current Biology*.

[62] Blattner F. R., Plunkett G., Bloch C. A. (1997). The complete genome sequence of *Escherichia coli* K-12. *Science*.

[63] Rubin B. E., Wetmore K. M., Price M. N. (2015). The essential gene set of a photosynthetic organism. *Proceedings of the National Academy of Sciences of the United States of America*.

[64] Wang Z., Wu M. (2015). An integrated phylogenomic approach toward pinpointing the origin of mitochondria. *Scientific Reports*.

[65] Spang A., Saw J. H., Jorgensen S. L. (2015). Complex Archaea that bridge the gap between prokaryotes and eukaryotes. *Nature*.

[66] Peng K., Radivojac P., Vucetic S., Dunker A. K., Obradovic Z. (2006). Length-dependent prediction of protein intrinsic disorder. *BMC Bioinformatics*.

[67] Schad E., Tompa P., Hegyi H. (2011). The relationship between proteome size, structural disorder and organism complexity. *Genome Biology*.

[68] Boc A., Diallo A. B., Makarenkov V. (2012). T-REX: a web server for inferring, validating and visualizing phylogenetic trees and networks. *Nucleic Acids Research*.

[69] Tamura K., Peterson D., Peterson N., Stecher G., Nei M., Kumar S. (2011). MEGA5: molecular evolutionary genetics analysis using maximum likelihood, evolutionary distance, and maximum parsimony methods. *Molecular Biology and Evolution*.

