# Supplementary material for: Classification of Complete Proteomes of Different Organisms and Protein Sets Based on Their Protein Distributions in Terms of Some Key Attributes of Proteins
Source: Int J Genomics. 2018 Mar 4;2018:9784161. doi: 10.1155/2018/9784161 (PMC5857298; doi:10.1155/2018/9784161)
Supplement: Supplementary Materials — Table S1. Correlation coefficients between ln(L) and ID%. Table S2. Intervals that partition the LD spaces into M × N blocks with M = N = 2 and 5. Table S3. IDPs in the mitochondrion and chloroplast of A. thaliana. Figure S1. Protein-density contour maps (see Figure 1(a) in main text for the scale bar). Figure S2. Phylogenetic trees reconstructed from the protein distributions in the LD space using A—(M = N = 2) and B (M = N = 5). Eukaryotes are in red, prokaryotes (bacteria and Archaea) in blue, and giruses in pink branches. MEGA5 (1) was used to plot the trees. Compared to that of the M = N = 10 tree (Figure 5), the branch length of the M = N = 10 tree is larger. Figure S3. Phylogenetic tree reconstructed from gene densities on the LD space. Different versions (v01–v03) of the P. trichocarpa proteomes have been used. By default of the present work, only proteins from primary transcripts are chosen for all proteomes. Here, for P. trichocarpa proteome v03, we tested both the primary transcripts (41,434 proteins) and all transcripts (73,013 proteins). We show here that progressive improvements including the splicing variants did not make significant changes in the phylogeny. [file 9784161.f1.pdf]

Supplementary Materials for

Classification of Complete Proteomes of Different Organisms and Protein Sets Based on Their Protein Distributions in Terms of Some Key Attributes of Proteins

Hao-Bo Guo[1], Yue Ma[1], Gerald A. Tuskan[2], Xiaohan Yang[1,2,*], Hong Guo[1,*]

[1]Department of Biochemistry and Cellular and Molecular Biology, University of Tennessee, Knoxville, TN 37996

[2]Biosciences Division, Oak Ridge National Laboratory, Oak Ridge, TN 37831

*Corresponding authors: HG (hguo1@utk.edu) or XY (yangx@ornl.gov)

The supplementary information include:

Table S1. Correlation coefficients between ln(*L*) and ID%
Table S2. Intervals that partition the *L-D* spaces into M×N blocks with M=N=2 and 5
Table S3. IDPs in the mitochondrion and chloroplast of *A. thaliana*
Figure S1. Protein-density contour maps
Figure S2. Phylogenetic trees reconstructed from the protein distributions in the *L-D* space using (**A**). M=N=2 and (**B**). M=N=5. Eukaryotes are in red, prokaryotes (bacteria and Archaea) in blue and giruses in pink branches, respectively. MEGA5 (*1*) was used to plot the trees. Compared to the M=N=10 (Fig. 4), the branch length of the tree is larger than the M=N=10 tree.
Figure S3. Phylogenetic tree reconstructed from gene densities on the *LD* space. Different versions (v01-v03) of the *P. trichocarpa* proteomes have been used. By default of the present work only proteins from primary transcripts are chosen for all proteomes. Here for *P. trichocarpa* proteome v03, we tested both the primary transcripts (41,434 proteins) and all transcripts (73,013 proteins). We show here that progressive improvements and including of the splicing variants did not make significant changes in the phylogeny.

Table S1. Correlation coefficients between ln(*L*) and ID.

Species[a]	Pearson	Spearman
C. reinhardtii	0.225	0.262
D. melanogaster	0.073	0.09
Monocercomonoides	0.052	0.047
H. sapiens	0.021	0.062
S. cerevisiae	-0.034	0.009
P. patens	-0.076	-0.049
P. trichocarpa	-0.084	-0.072
Mimivirus	-0.125	-0.155
A. thaliana	-0.154	-0.138
G. intestinalis	-0.176	-0.146
Viruses (gene set)	-0.189	-0.17
A. comosus	-0.201	-0.187
A. trichopoda	-0.222	-0.209
Rickettsiales	-0.224	-0.208
Pandoravirus	-0.244	-0.221
Plasmids (gene set)	-0.25	-0.212
S. elongatus	-0.268	-0.23
I. hospitalis	-0.317	-0.254
O. sativa	-0.328	-0.309
N. equitans	-0.348	-0.251
Plastids (gene set)	-0.357	-0.439
E. coli	-0.358	-0.287
Lockiarchaeum	-0.375	-0.322
Mitochondria (gene set)	-0.507	-0.468
All proteins[b]	-0.101	-0.129
p-value[c]	<2.2e-16	<2.2e-16

a. Ranked by the Pearson's CC. Also see Table 1 in main text;
b. All 811,600 protein entries studied in this work
c. P-values estimated using the program R

Table S2. Intervals that partition the *L-D* spaces into M×N blocks with M=N=2 and 5

2×2	**#**	*1*	*2*
	**ln(*L*)**	[0,5.8)	[5.8, ∞)
	L	[1,331)	[331, ∞)
	**ID%**	[0,0.5)	[0.5,1.0]

5×5	**#**	**1**	**2**	**3**	**4**	**5**
	**ln(*L*)**	[0,4.9)	[4.9,5.5)	[5.5,6.1)	[6.1,6.7)	[6.7, ∞)
	L	[1,135)	[135,245)	[245,446)	[446,813)	[813, ∞)
	**ID%**	[0,0.2)	[0.2,0.4)	[0.4,0.6)	[0.6,0.8)	[0.8,1.0)

Table S3. IDPs in the mitochondrion and chloroplast of *A. thaliana*

Organelle	Gene	Length (aa)	ID%	Annotation[a]
Mitochondrion	ATMG00890	106	100	Cytochrome C assembly protein (ORF106D)
	ATMG00200	107	100	Unknown
	ATMG00660	149	100	Unknown
	ATMG00980	125	96.8	Ribosomal protein L2 (RPSL2)
	ATMG01290	111	95.5	Unknown
	ATMG00880	187	75.9	Unknown
	ATMG01330	127	74.8	Unknown
	ATMG01130	106	72.6	Unknown
	ATMG01030	106	71.7	Unknown
	ATMG00130	121	68.6	Unknown
	ATMG01040	107	68.2	Unknown
	ATMG00010	153	68.0	Unknown
	ATMG00870	184	66.8	Unknown
	ATMG00670	275	60.7	Unknown
	ATMG00540	122	59.0	Unknown
	ATMG00560	349	55.6	Ribosomal protein L2 (RPL2)
	ATMG01010	118	52.5	Unknown
	ATMG00690	240	52.5	Unknown
	ATMG01100	105	52.4	Unknown
	ATMG01400	105	52.4	Unknown
	ATMG00140	167	52.1	Unknown
	ATMG00910	215	52.1	Unknown
	ATMG01200	294	50.0	ATPase F1 complex, alpha subunit protein (ORF294)
Chloroplast	ATCG00760	37	100	Ribosomal protein L36 (RPL36)
	ATCG01020	52	100	Ribosomal protein L32 (RPL32)
	ATCG00330	100	74.0	Ribosomal protein S14 (RPS14)
	ATCG00650	101	70.3	Ribosomal protein S18 (RPS18)
	ATCG01120	88	69.3	Ribosomal protein S15 (RPS15)
	ATCG00660	117	50.4	Ribosomal protein L20 (RPL20)

a. Annotations obtained from The Arabidopsis Information Resource (TAIR).

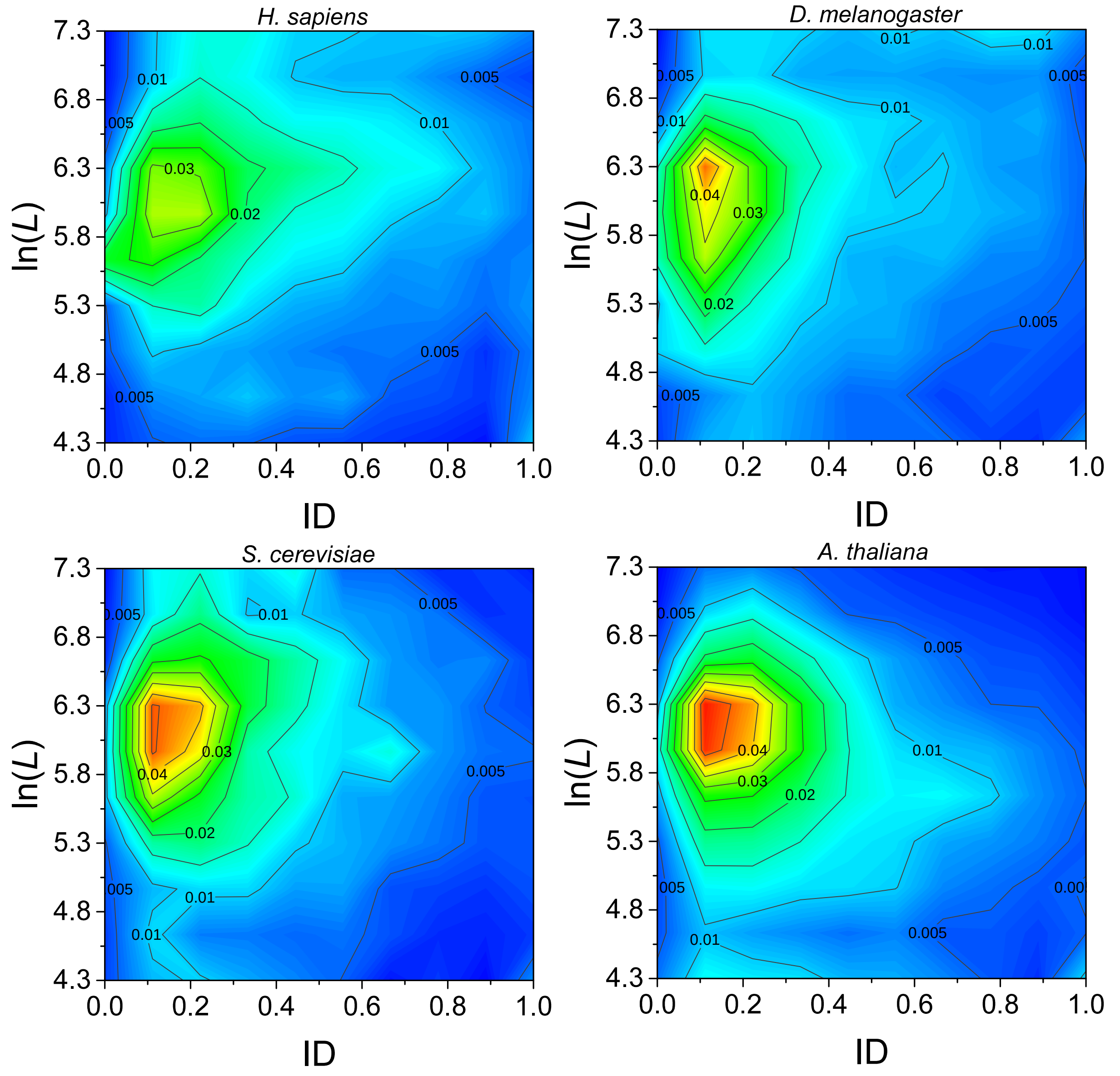

Figure S1. Protein-density contour maps (See Figure 3A in main text for the scale bar).

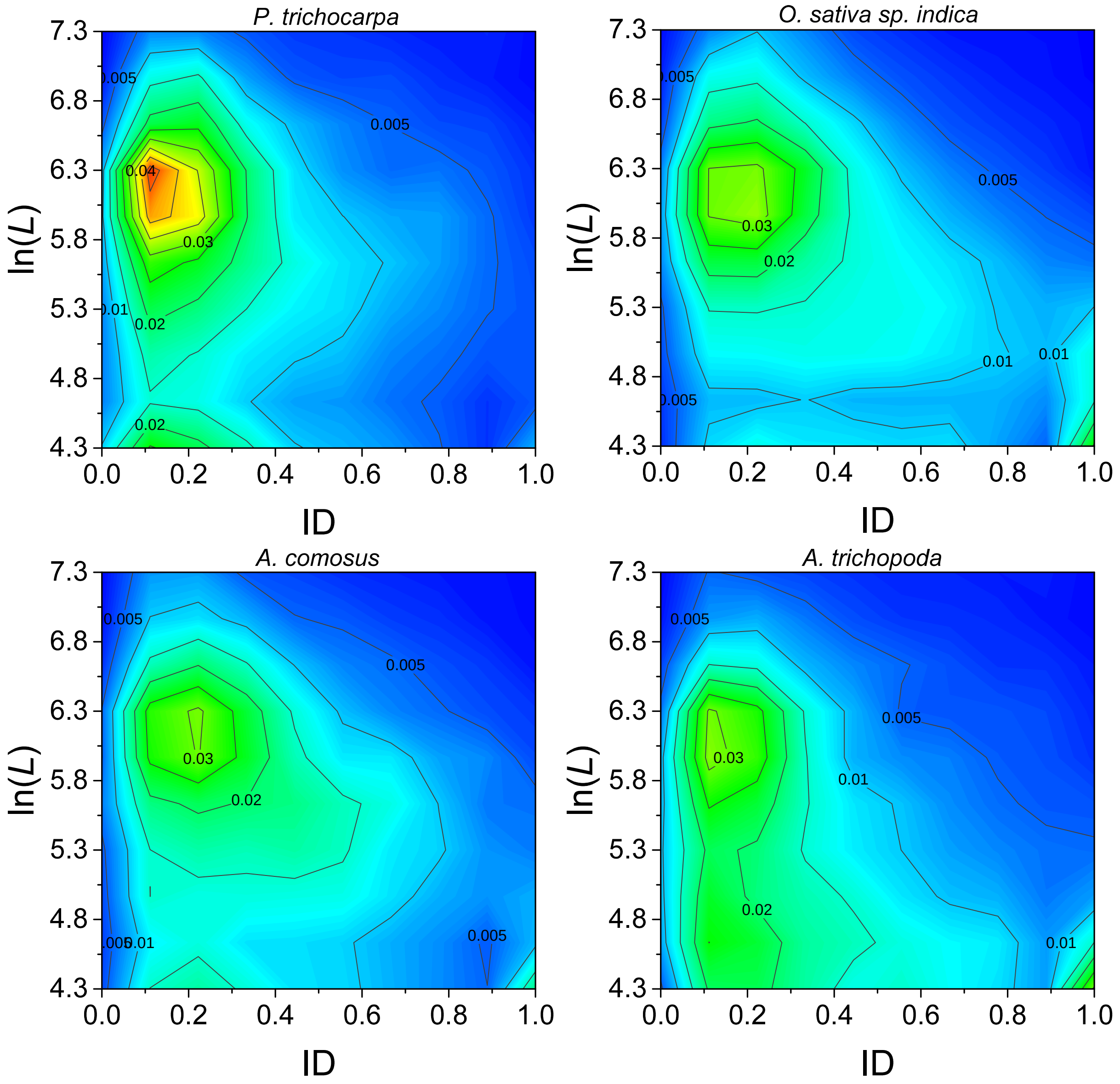

Figure S1 (continued). Protein-density contour maps, continued (See Figure 3 in main text for the scale bar).

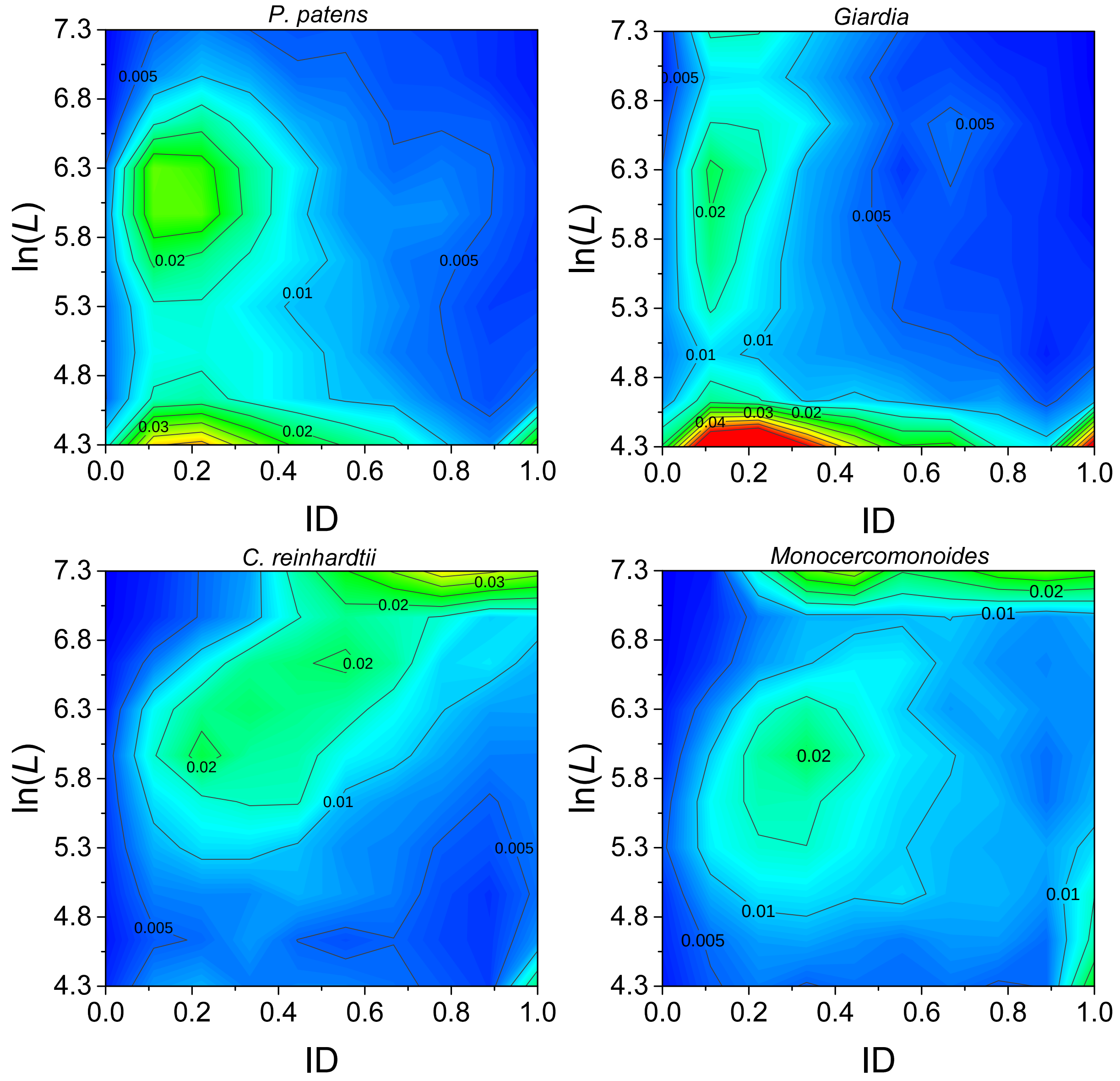

Figure S1 (continued). Protein-density contour maps, continued (See Figure 3 in main text for the scale bar).

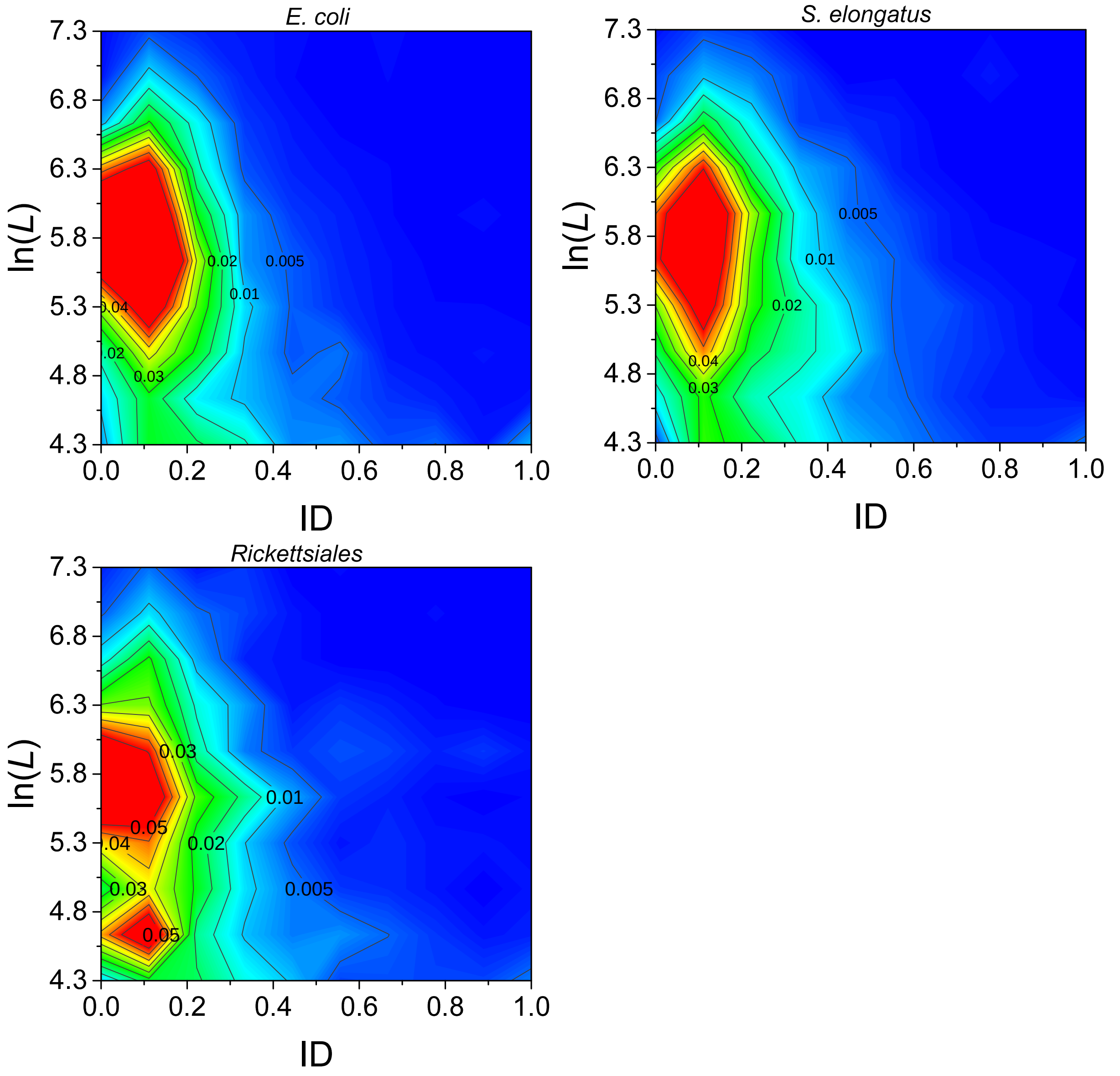

Figure S1 (continued). Protein-density contour maps, continued (See Figure 3 in main text for the scale bar).

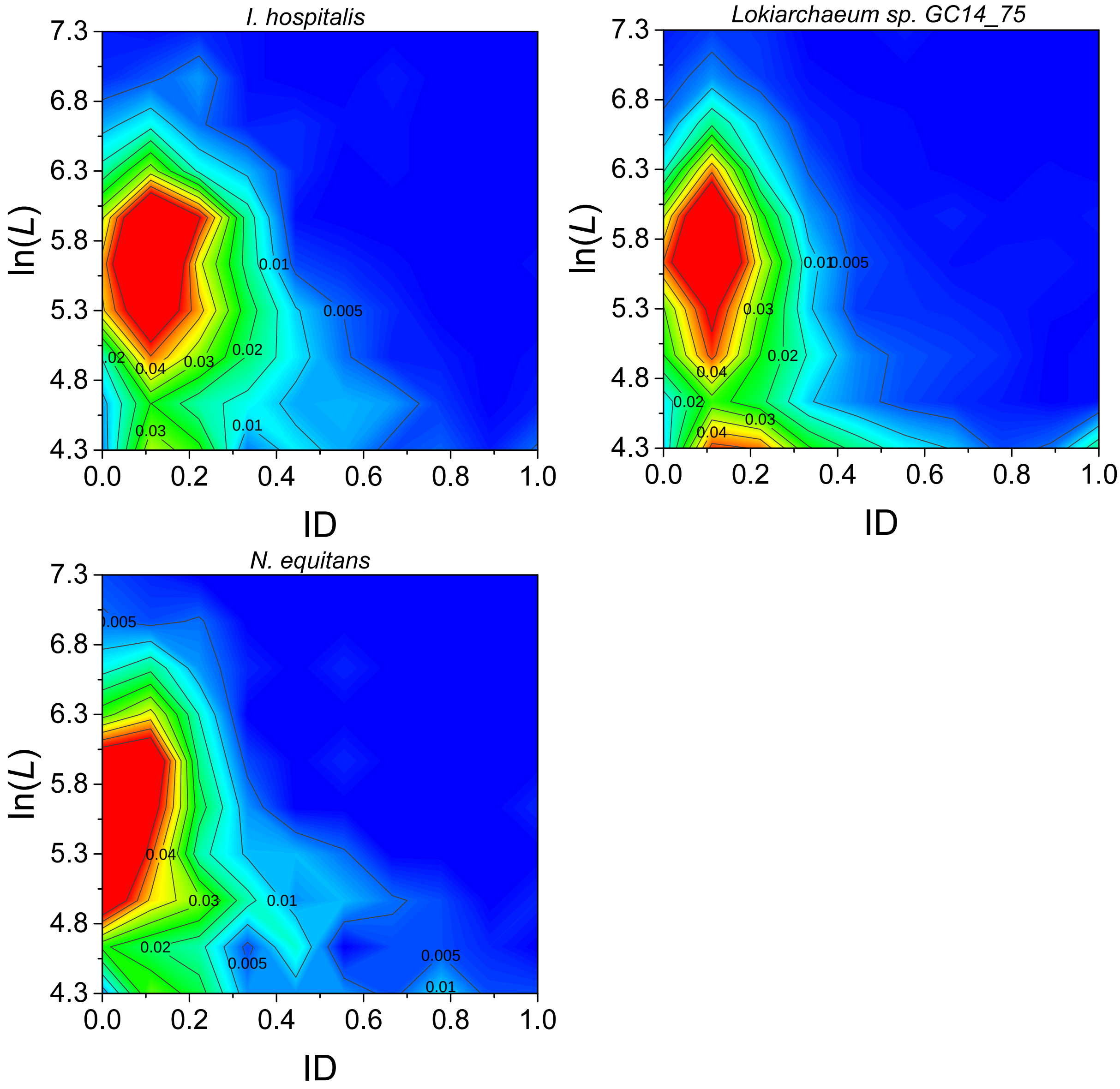

Figure S1 (continued). Protein-density contour maps, continued (See Figure 3 in main text for the scale bar).

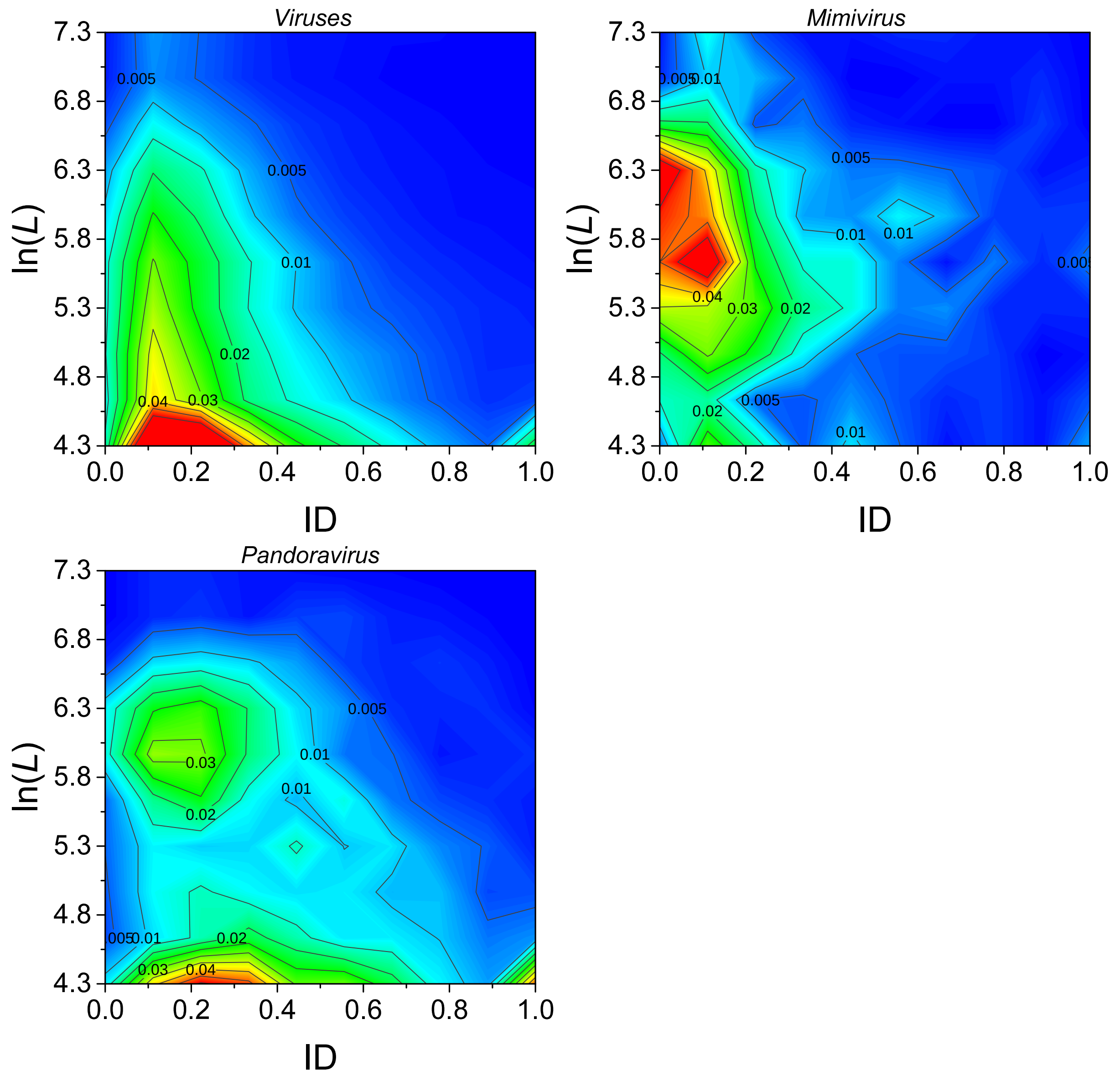

Figure S1 (continued). Protein-density contour maps, continued (See Figure 3 in main text for the scale bar).

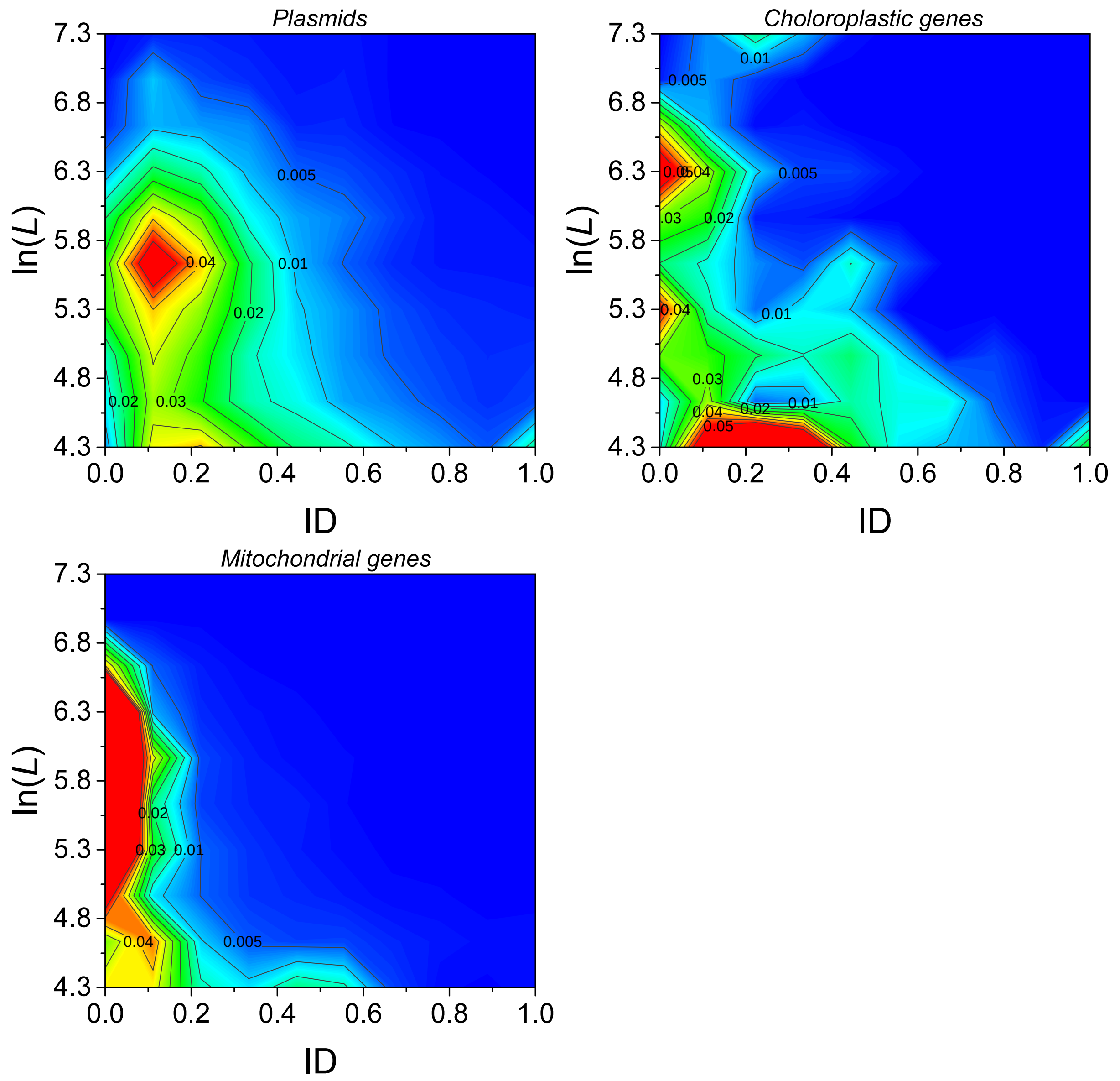

Figure S1 (continued). Protein-density contour maps, continued (See Figure 3 in main text for the scale bar).

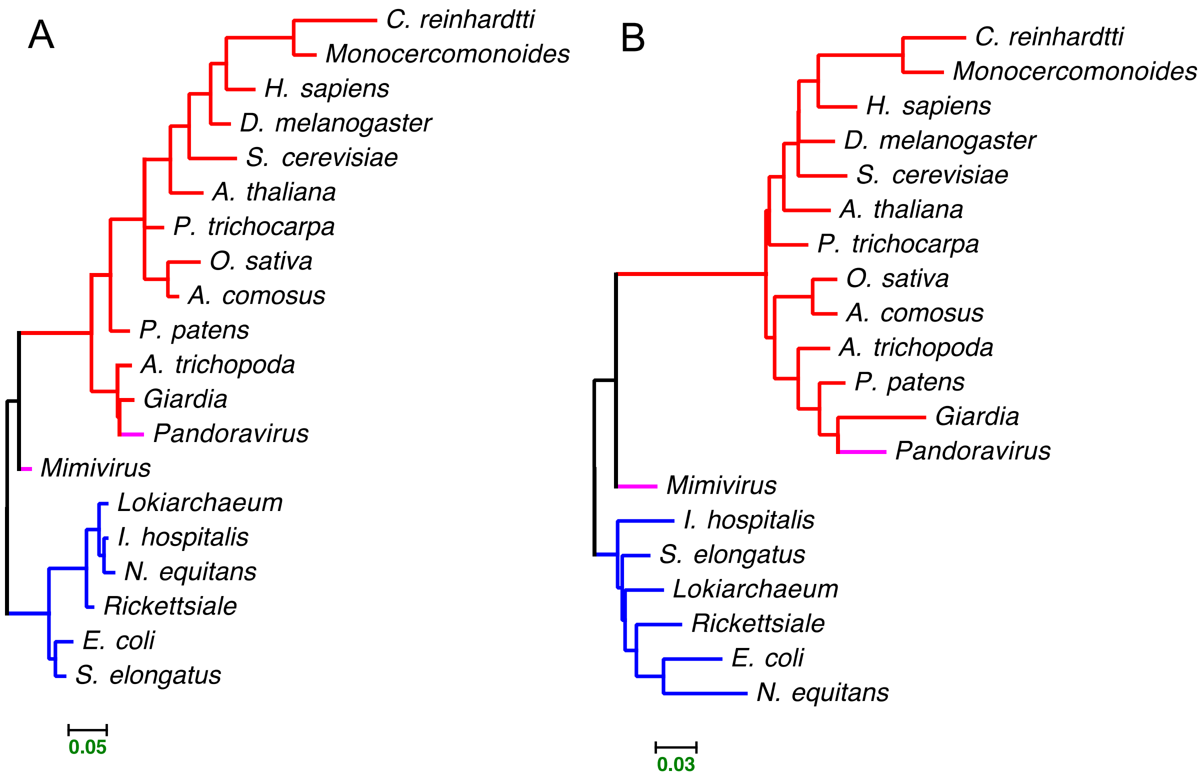

Figure S2. Phylogenetic trees reconstructed from the protein distributions in the *L*-*D* space using (**A**). M=N=2 and (**B**). M=N=5. Eukaryotes are in red, prokaryotes (bacteria and Archaea) in blue and giruses in pink branches, respectively. MEGA5 (*1*) was used to plot the trees. Compared to the M=N=10 (Fig. 4), the branch length of the tree is larger than the M=N=10 tree.

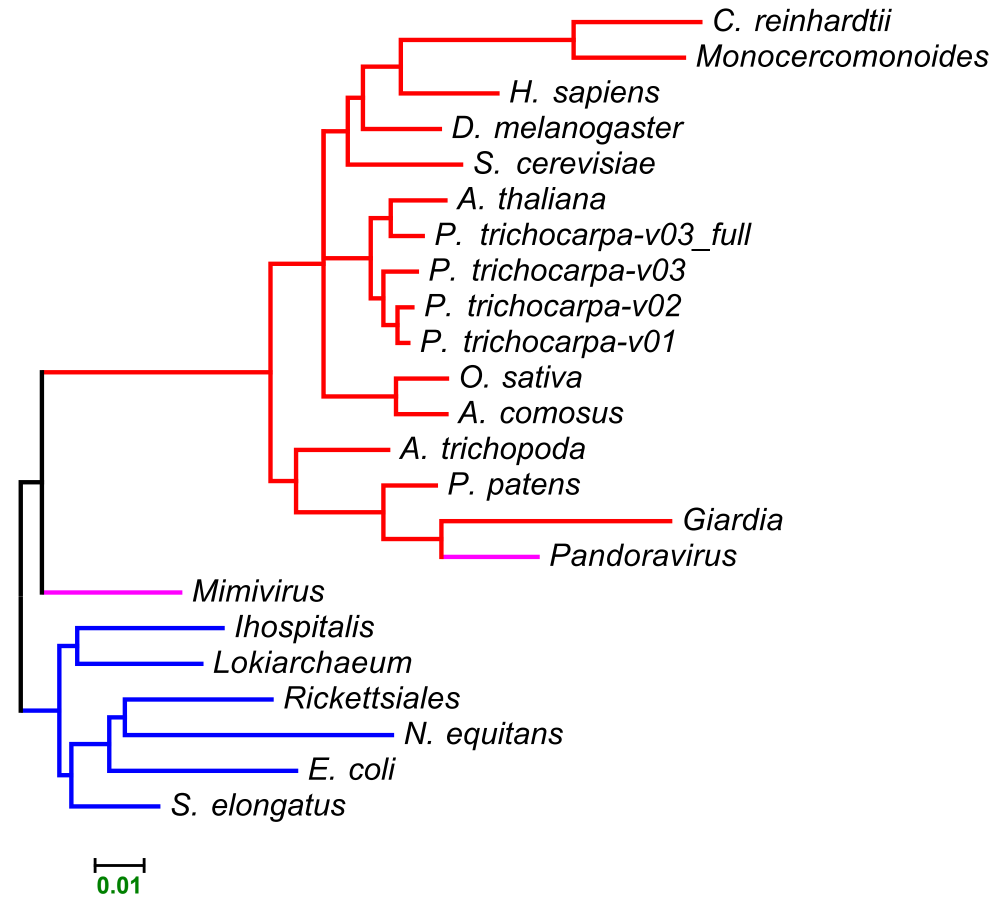

Figure S3. Phylogenetic tree reconstructed from gene densities on the *LD* space. Different versions (v01-v03) of the *P. trichocarpa* proteomes have been used. By default of the present work only proteins from primary transcripts are chosen for all proteomes. Here for *P. trichocarpa* proteome v03, we tested both the primary transcripts (41,434 proteins) and all transcripts (73,013 proteins). We show here that progressive improvements and including of the splicing variants did not make significant changes in the phylogeny.

References

1. K. Tamura *et al.*, MEGA5: Molecular Evolutionary Genetics Analysis Using Maximum Likelihood, Evolutionary Distance, and Maximum Parsimony Methods. *Mol Biol Evol* **28**, 2731 (Oct, 2011).
